# A Broad Temperature Active Lipase Purified From a Psychrotrophic Bacterium of Sikkim Himalaya With Potential Application in Detergent Formulation

**DOI:** 10.3389/fbioe.2020.00642

**Published:** 2020-06-25

**Authors:** Anil Kumar, Srijana Mukhia, Neeraj Kumar, Vishal Acharya, Sanjay Kumar, Rakshak Kumar

**Affiliations:** ^1^Biotechnology Division, CSIR-Institute of Himalayan Bioresource Technology, Palampur, India; ^2^Academy of Scientific and Innovative Research (AcSIR), CSIR-Institute of Himalayan Bioresource Technology, Palampur, India; ^3^Department of Microbiology, Guru Nanak Dev University, Amritsar, India

**Keywords:** *Chryseobacterium polytrichastri* ERMR1:04, lipase, purification, broad temperature activity, bioinformatics analysis, detergent formulation

## Abstract

Bacterial lipases with activity spanning over a broad temperature and substrate range have several industrial applications. An efficient enzyme-producing bacterium *Chryseobacterium polytrichastri* ERMR1:04, previously reported from Sikkim Himalaya, was explored for purification and characterization of cold-adapted lipase. Optimum lipase production was observed in 1% (v/v) rice bran oil, pH 7 at 20°C. Size exclusion and hydrophobic interaction chromatography purified the enzyme up to 21.3-fold predicting it to be a hexameric protein of 250 kDa, with 39.8 kDa monomeric unit. MALDI-TOF-MS analysis of the purified lipase showed maximum similarity with alpha/beta hydrolase (lipase superfamily). Biochemical characterization of the purified enzyme revealed optimum pH (8.0), temperature (37°C) and activity over a temperature range of 5–65°C. The tested metals (except Cu^2+^ and Fe^2+^) enhanced the enzyme activity and it was tolerant to 5% (v/v) methanol and isopropanol. The Km and Vmax values were determined as 0.104 mM and 3.58 U/mg, respectively for *p*-nitrophenyl palmitate. Bioinformatics analysis also supported *in vitro* findings by predicting enzyme's broad temperature and substrate specificity. The compatibility of the purified lipase with regular commercial detergents, coupled with its versatile temperature and substrate range, renders the given enzyme a promising biocatalyst for potential detergent formulations.

## Introduction

Lipases include an important group of biocatalysts that belong to the class of triacylglycerol hydrolases (EC: 3.1.1.3) (Gupta et al., 2004). They break down fats and are highly active against water-insoluble substrates. The lipolytic enzymes are a part of serine hydrolase superfamily whose activities depend on the catalytic triad of Ser, His, and Asp residues (Brumlik and Buckley, 1996). In addition to hydrolyzing carboxylic acid esters, they can perform esterification and transesterification reactions (Reetz, 2002). Lipases have high enantioselectivity with the ability to perform in aqueous and non-aqueous environments which makes them highly suitable biocatalysts in many industries (Kumar et al., 2016a).

Lipases are ubiquitous as they are found in all groups of organisms from bacteria to plants and animals. Although lipases have been studied widely, not much attention has been paid to the bacterial lipases from extreme cold environments (Salwoom et al., 2019b). Lipases from high altitude bacteria are of significance due to the host of specific structural features that these enzymes have evolved as a mode of adaptation to frequent freezing and thawing conditions. They find potential industrial applications in diverse fields of detergent additives, food processing, environmental bioremediation in cold, and many more (Maiangwa et al., 2015). High-temperature active lipases are suitable biocatalysts in the synthesis of biopolymers, pharmaceutical compounds, and cosmetics owing to their thermostability and organic solvent tolerance (Ranjan et al., 2018). Due to the inactivation of enzyme components at low temperature, detergents lose their functionality. Low-temperature active lipases are greatly advantageous as detergent additives for cold washing that would diminish energy consumption as well as wear and tear of delicate fabrics (Joseph et al., 2008).

Several low-temperature, cold-adapted environments like high-altitude mountains, glaciers, and deep seas serve as sources of psychrophilic and psychrotrophic microorganisms. Psychrophilic microorganisms are cold-loving with a minimum, optimum, and maximum temperature for growth at about 0, 15, and 20°C, respectively, while psychrotrophic organisms have growth maxima above 25°C but can survive in low temperature (Junge et al., 2019). Cold-active enzymes play a major role in their physiological adaptation to low temperature and have been a focus of research at recent times owing to their potential applications in biotechnological industries (Salwoom et al., 2019b). Irrespective of their significance, cold-active lipases have some limitations like diminished activity at moderate to high temperature, limited substrate specificity, and less thermostability (Joseph et al., 2008; Kavitha, 2016). As temperature-related activity is a major characteristic for the use of enzymes in industry, lipases produced by psychrotolerant bacteria with a wider range of temperature activity can be preferable.

East Rathong glacier in Sikkim Himalaya is an unexplored region to obtain extremophilic microbial resources for bioprospection of industrial enzymes. The debris-free valley glacier experiences cold and wet climate throughout and snowfall is common even during monsoon (Luitel et al., 2012). We have reported genome-based predictions on cold-active enzymes from the psychrotrophic bacteria thriving in Sikkim Himalaya (Kumar et al., 2015a, 2016b, 2018a, 2019; Himanshu et al., 2016). In the current study, one such bacterium *Chryseobacterium polytrichastri* ERMR1:04 reported as a potential candidate for various industrial enzymes has been explored (Kumar et al., 2015b). The isolate showed promising results for lipase activity in plate assay with clear halo zone indicating tributyrin hydrolysis. Considering the significance of lipase from high-altitude bacteria, we isolated, purified and characterized the enzyme in the present study. Bioinformatics verification of the *in vitro* findings was carried out using amino acid sequence-based analysis for prediction of enzyme structure, and its behavior over different temperature and substrates. Finally, to determine the appropriate application of the new enzyme from Sikkim Himalaya, we tested its compatibility with a range of commercially available detergents at different temperature for its suitability as a detergent formulation.

## Materials and Methods

### Chemicals

Following analytical grade chemicals were used in the current study: *p*NPP (*para*-nitrophenyl palmitate), Sephadex G-100, Octyl-Sepharose fast flow, were purchased from Sigma-Aldrich (St. Louis, MO, USA). Bradford solution, ammonium sulfate, triton X-100, SDS were purchased from Himedia Laboratories, Mumbai India.

### Bacterial Strain

A previously isolated bacterial strain *Chryseobacterium polytrichastri* ERMR1:04 was used in the present study (Kumar et al., 2015b).

### Optimization of Culture Conditions and Production of Extracellular Lipase

Culture conditions like temperature, pH, substrate type, and substrate concentration were optimized to maximize lipase production by the bacterial isolate. The bacterium was grown in production broth containing (g/L) yeast extract (5.0), potassium chloride (0.50), sodium nitrate (3.0), ferrous sulfate heptahydrate (0.01), dipotassium hydrogen phosphate (0.10), magnesium sulfate heptahydrate (0.50) and rice bran oil (1% v/v) (Sharma et al., 2018). The seed culture was prepared in ABM broth containing (g/L) Peptone (5) and Yeast extract (2) (Shivaji et al., 2013). About 1% (v/v) of overnight grown seed culture was transferred in previously described production broth and kept at 150 rpm for 60 h. Firstly, the *C. polytrichastri* ERMR1:04 was allowed to grow in different vegetable oils, i.e., olive oil, rice bran oil, sapium oil, and sunflower oil. The broth showing maximum lipase activity was selected and then bacterial isolate was grown at different temperature of 10, 15, 20, 28°C. Bacterial isolate was also grown at different pH of 6–9. The effect of different oil concentration on the production of lipase by the isolate in the culture broth was checked by growing it in 0.5,1, 1.5, 2, and 2.5% (v/v) vegetable oil.

### Lipase Activity Determination

Lipase activity was determined colourimetrically using the substrate *p*NPP by slight modification in a previous method (Zhang et al., 2018). In detail, 40 μL of 5 mM *p*NPP substrate dissolved in isopropanol was mixed with 900 μL of 50 mM Tris buffer pH-8 containing 0.3% (v/v) Triton X-100. The reaction was initiated by adding 60 μL of culture supernatant and incubating the reaction tubes at 20°C for 10 min. After incubation, the reaction was stopped by placing the reaction tubes at (−20°C) for 10 min. Reaction control without enzyme was also run in parallels. The amount of *para*-nitrophenol released after completion of the reaction was calculated by checking the absorbance at 410 nm using *para*-nitrophenol as standard. All reactions were done in triplicates. One unit of lipase activity was defined as the amount of enzyme required to release 1-μmol of *para*-nitrophenol from substrate per minute at standard assay condition. Protein estimation was done by the method of Bradford (Bradford, 1976) with bovine serum albumin as standard.

### Purification of *C. polytrichastri* ERMR1:04 Extracellular Lipase

The cell-free supernatant containing the extracellular lipase was collected by centrifuging the bacterial culture broth at 12,000 × g for 10 min at 4°C. To concentrate the protein, solid ammonium sulfate was added until 70% saturation was reached. After overnight precipitation in stirring condition at 4°C, the precipitated proteins were collected by centrifuging at 15,000 × g for 20 min at 4°C and dissolved in 50 mM Tris buffer pH 8. Dialysis was done at 4°C with a dialysis membrane (with a cut-off of 10 kDa) to remove the salt ions from protein samples with three changes in the buffer every 4 h. The dialyzed protein sample was then further purified using size exclusion and hydrophobic interaction chromatography. The purified lipase was stored at 4°C until further use.

### Size Exclusion Chromatography

A pre-swollen Sephadex G-100 column was equilibrated with 50 mM Tris HCl buffer pH 8. Dialyzed protein sample was clarified by passing through a 0.45 μm syringe filter before loading onto the column. Protein volume of 2 mL was loaded onto the column. Elution was done in the same buffer at a flow rate of 0.5 mL/min. The eluted fractions were checked for protein content and lipase activity and then pooled, concentrated by Amicon centrifugal filters (10 kDa cut-off), and subjected to further purification.

### Hydrophobic Interaction Chromatography

An Octyl-Sepharose fast flow column was equilibrated with 50 mM Tris HCl buffer pH 8 containing 1 M ammonium sulfate. Sephadex G-100 purified protein fraction was loaded (2 mL) onto the column. The elution was done with a linear gradient of ammonium sulfate from 1 to 0 M in 50 mM Tris HCl buffer pH 8 at a flow rate of 0.5 mL/min. The fractions having the lipase activity were pooled and then dialyzed against 50 mM Tris HCl buffer pH 8 and checked for its purity and molecular weight analysis by SDS-PAGE.

### Determination of Molecular Weight of Purified Lipase and Zymography

SDS-PAGE (12%) and Native PAGE (10%) analysis were done to check the purity and molecular weight of purified bacterial lipase (Laemmli, 1970). Zymogram analysis of purified lipase was performed using MUF-butyrate as substrate according to a previously described method (Prim et al., 2003).

### Peptide Mass Fingerprinting by MALDI-TOF-MS

The gel bands were manually excised and then de-stained with 40 mM ammonium bicarbonate solution followed by dehydration with acetonitrile. In-gel modification of cysteine residues was done with 10 mM dithiothreitol and 55 mM iodoacetamide. The proteins in the bands were then subjected to digestion with trypsin enzyme, followed by overnight incubation at 37°C. Trypsin digested samples were then mixed with 0.1% trifluoroacetic acid and sonicated in a bath sonicator for 5–10 min. The protein solution obtained was then mixed with an equal volume of cyno-4-hydroxycinnamic acid matrix and loaded onto the MALDI plate and analyzed by Bruker Autoflex MALDI-TOF-MS (Bruker Daltonics) system. The mass profile of peptides obtained was then compared with a MASCOT database.

*C. polytrichastri* ERMR1:04 draft genome was then searched (from NCBI protein database) for lipase and its related amino-acid sequences. The protein sequence, alpha/beta hydrolase (topmost hit in MALDI-TOF-MS) was then aligned with the ERMR1:04 lipase and its related protein sequences (Kumar et al., 2015b) using ClustalW multiple sequence alignment tool. The phylogenetic tree was constructed using the neighbor-joining method in MEGA-X software based on 1,000 bootstrap replications (Kumar et al., 2018b). The protein sequence showing maximum similarity with the ERMR1:04 protein was then used for the bioinformatics analysis.

### Biochemical Characterization of Purified Lipase

#### Effect of Temperature, pH, and Metal Ions

To check the effect of temperature and pH on purified lipase, activity was performed at a temperature ranging from 5 to 65°C and pH range of (7–9) using *p*NPP as substrate and 50 mM Tris buffer. To determine the effect of various metal ions (Na^+^, K^+^, Ca^2+^, Mg^2+^, Cu^2+^, Mn^2+^, Fe^2+^) on purified lipase, the enzyme was incubated with 5 and 10 mM salts for 30 min at 37°C. After incubation, the enzyme activity was checked at 37°C using *p*NPP as substrate.

#### Effect of Organic Solvents, Inhibitors, and Kinetic Study of Purified Lipase

The effect of common organic solvents on purified lipase was determined by incubating lipase in 5% (v/v) solvents (Butanol, Isopropanol, Acetonitrile, and Methanol) for 30 min at 37°C. The effect of various inhibitors (EDTA, DTT, PMSF) on enzyme activity was determined by incubating the enzyme in 1, 5, and 10 mM inhibitors at 37°C for 30 min in 50 mM Tris buffer pH 8. The lipase assay was performed using *p*NPP as a substrate in 50 mM Tris buffer pH 8 at 37°C for 10 min. The kinetic parameters (K_m_ and V_max_) were studied by varying *p*NPP concentration from 0.025 to 0.3 mM according to Lineweaver-Burk plots at 37°C (Lineweaver and Burk, 1934).

#### Detergent Compatibility Test

Detergent compatibility tests were conducted by using 1% (v/v) commercially available detergents, i.e., Tide, Surf, Aerial, and Wheel. Detergent solutions were prepared in distilled water and hydrolytic activity was checked in *p*NPP. The hydrolytic activity of lipase in the presence of commercial detergents was taken as a test while lipase activity in the absence of detergent was taken as control. The assay was done at different temperature (5, 20, 37, 45, and 60°C) for 10 min.

### Bioinformatics Analysis of Lipase Sequence

#### Protein Modeling

Protein sequences whose identity was found to be the highest with the related *C. polytrichastri* ERMR1:04 lipase sequence were considered and various physicochemical parameters (amino acid composition, molecular weight, Isoelectric point, GRAVY index etc.) were calculated for a sequence using ProtParam (https://web.expasy.org/protparam/) module implied in the ExPASy tools repository (https://www.expasy.org/tools/). The domain and motif analysis of the protein query was carried out with NCBI-CDD (Berman et al., 2000) repository.

Homology modeling is a technique deployed to predict or build a 3D structure of a protein with the help of template structures, which has good sequence similarity with the query sequence. The query protein sequence has a very low percentage similarity when subjected to Protein Data Bank (PDB) (Berman et al., 2000) database for template selection in Protein BLAST (Altschul et al., 1990). We proposed to find a better suitable template for our modeling purpose by deploying Modeler v9.20 (Webb and Sali, 2016) with multiple templates found via PSI-BLAST (Ye et al., 2006). The models built by modeler were assessed using the DOPE score and Z-score (Zhang and Skolnick, 1998) by default and then were validated employing different software *viz*. ProSAweb server (https://prosa.services.came.sbg.ac.at/prosa.php), PROCHECK (a standalone package) (Laskowski et al., 1993) and QMEAN package (Benkert et al., 2011). Further, energy minimization of the selected model was carried out using GROMACS 2018 (Abraham et al., 2015), to improve its stereochemistry. After we finally obtained a valid 3D model, we further exploited the structure with various tools like RaptorX (http://raptorx.uchicago.edu/StructPredV2/predict/) and COACH (a standalone package) (Yang et al., 2014) to find its binding site and evaluated its conserved domains.

#### Molecular Docking Simulation

Molecular docking simulation is used to predict the interaction pattern between the active site of protein and ligand. The docking process includes two steps, (a) assessment of conformation pattern of the ligand in the active site of the protein, (b) predicting binding affinity between the binding site and appropriate confirmation of ligand. For optimization of substrates for lipase production, a range of vegetable oils were tested which contain a different proportion of linoleic acid, palmitic acid, and oleic acid. These fatty acids act as inducers for lipase production. The significance of these fatty acids (linoleic acid, palmitic acid, and oleic acid) as inducers was checked by docking study. Effect of various substrates on lipolytic activity was also checked to know the nature of the enzyme through docking of various substrates *viz para*-nitrophenyl acetate, *para*-nitrophenyl palmitate, and *para*-nitrophenylphosphoryl choline. For docking simulation process, Autodock tools (Morris et al., 2009) and Autodock Vina (Trott and Olson, 2010) were used where it gave top eight poses as output and best pose was selected based on interaction and binding affinity. Pymol (DeLano, 2002) was further used to visualize and assess our docking results.

#### Molecular Dynamics Simulation (MDS)

Molecular dynamics simulation (MDS) is a computer-based graphics-intensive process used to analyze the physical movement of molecules at an atomistic level over a fixed course of the time and primarily used to understand the dynamic behavior of a biological structure. GROMACS v2018, an open-source tool, was employed to study the query protein in both ways (intact as well as in the presence of substrate). MDS was employed at various temperature to understand the broad temperature character of query lipase. Firstly, the intact protein was treated with OPLSS-AA force field, placed in the center of the cubic box, solvated and ions were added to maintain the neutrality of the overall system. The energy of the whole system was minimized with the steepest descent algorithm up to 50,000 steps. Further minimized system was followed with equilibration process using NVT and NPT ensembles for 100 picoseconds (ps) at various temperature, i.e., 268.15 K (−5°C), 278.15 K (5°C), 293.15 K (20°C), 310.15 K (37°C), 313.15 K (40°C), and 333.15 K (60°C) using Berendsen coupling. Final MDS was run for 5 nanoseconds (ns) with given temperature condition. LINC algorithm and particle mesh Ewald (PME) were employed during NVT, NPT, and final run to calculate bond constraints and long-range electrostatics treatment. The same protocol was used for MDS of lipase in complex with substrates, where GROMOS9654a7 force field was used to generate topology of the complex. PRODRG 2.5 webserver (http://prodrg1.dyndns.org/submit.html) was used to retrieve ligand topology. Final MDS was carried out for 5 ns at 278.15 K (5°C). All computational calculations were carried out on Ubuntu 16.104 Tyrone workstation supported with Intel Xeon Gold6132 and Quadro P5000 graphics architecture.

## Results

### Optimization of Culture Condition

**Figure 1:**
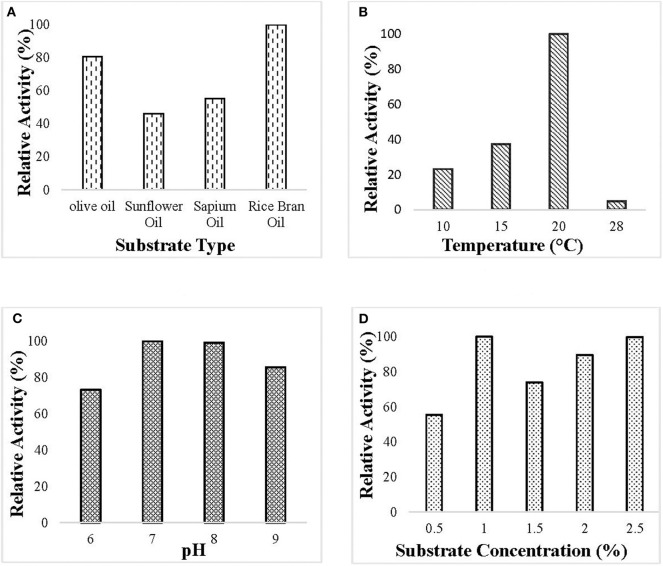
Optimization of culture conditions for the production of ERMR1:04 lipase. The bacterial isolate was grown in mineral-based production media for 60 h at 150 rpm. The optimized condition for lipase production was determined by checking the relative activity of the lipase produced at different experimental parameters, and the maximum was set to 100%. Lipase activity was determined using 50 mM tris buffer pH 8 at 37°C. Effect of **(A)** different substrate types (olive oil, sunflower oil, sapium oil, and rice bran oil) on the production of lipase. The bacterial isolate was grown at 20°C pH 7 having 1.5% (v/v) oil. Maximum lipase production was observed in rice bran oil; **(B)** different temperature (10, 15, 20, and 28°C) on lipase production in 1.5% (v/v) rice bran oil pH 7. Maximum lipase production was observed at 20°C; **(C)** different pH (6–9) on lipase production. The bacterial isolate was grown in 1.5% (v/v) rice bran oil at 20°C. Maximum lipase production was observed at pH 7; **(D)** different substrate percentage (0.5–2.5% v/v) on lipase production. The bacterial isolate was grown at 20°C pH 7 having rice bran oil as a substrate. Maximum lipase production was observed in 1% (v/v) rice bran oil.

Optimization of culture condition was done to maximize the production of the lipase. Maximum lipase activity was observed in 1% (v/v) rice bran oil followed by olive oil. Optimum lipase production was observed at pH 7 when the bacterial isolate was incubated at 20°C and 150 rpm for 55 h (Figure 1).

### Purification of Extracellular Lipase

**Figure 2:**
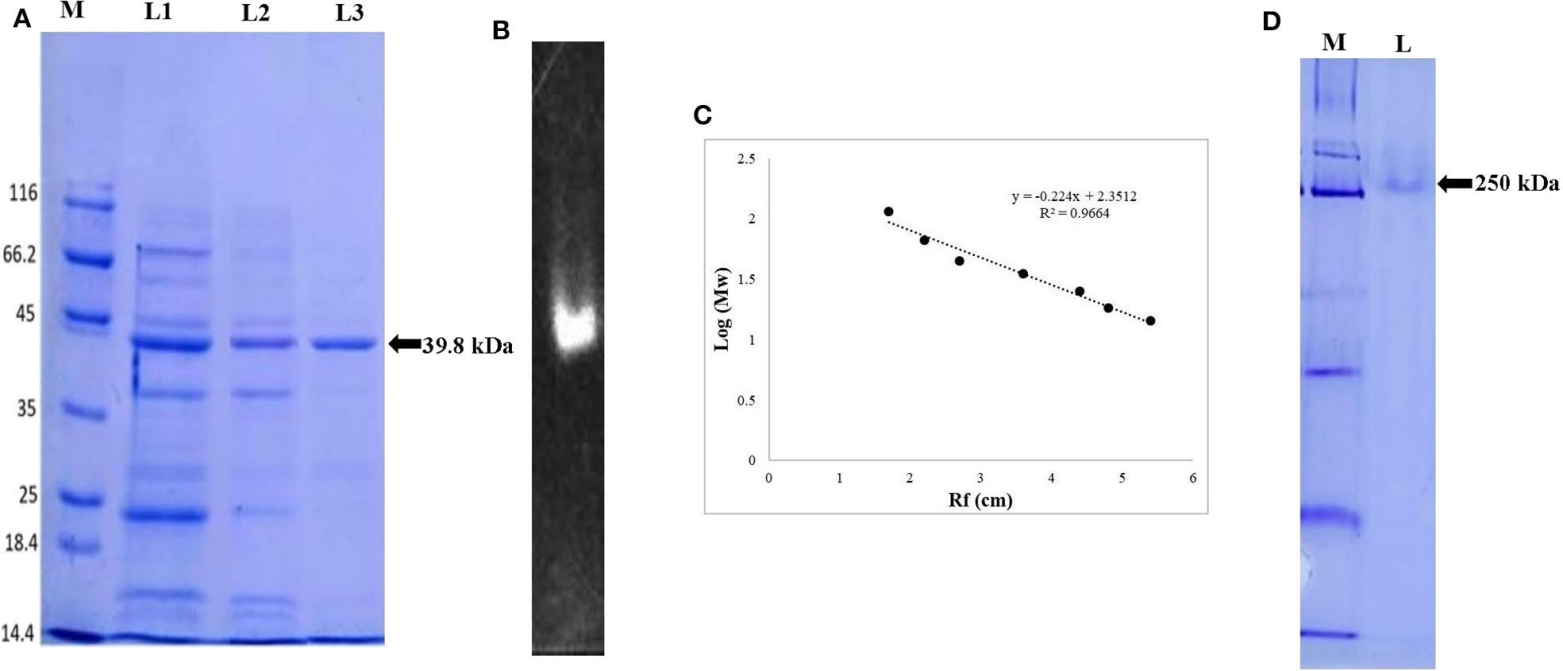
Electrophoretic pattern of lipase, **(A)** SDS-PAGE analysis of ERMR1:04 lipase at each purification step. The proteins were separated on 12% SDS-PAGE gel and then stained with Coomassie Blue R-250. M represents molecular weight marker (Pierce™ Unstained Protein MW Marker, Thermo Scientific); L1: crude cell-free extract of ERMR1:04; L2: size exclusion chromatography (Sephadex G-100) purified protein fraction; L3: hydrophobic interaction chromatography (Octyl-Sepharose fast flow) purified lipase; **(B)** zymographic analysis of purified lipase in the presence of substrate MUF-butyrate in 12% SDS-PAGE; **(C)** standard curve between log [MW] vs. Rf of proteins moved on SDS-PAGE (12%). The linear relationship between protein MW markers and migration distance showed reliability in predicting MW of lipase; **(D)** native-PAGE (10%) analysis of purified lipase. M represents the molecular weight marker; L represents hydrophobic interaction chromatography (Octyl-Sepharose fast flow) purified lipase fraction.

The extracellular ERMR1:04 lipase (3.4 U/mg, 0.088 mg/mL protein) secreted in 500 mL fermentation broth was subjected to ammonium sulfate precipitation up to 30–80% saturation followed by dialysis against 50 mM Tris buffer with 10 kDa dialysis membrane. The 30–80% dialyzed sample showed a maximum specific activity of 26.16 U/mg with 7-fold purification. Sephadex G-100 purified lipase showed a specific activity of 35 U/mg with 10-fold purification. The SDS-PAGE analysis of the G-100 purified fractions showed multiple bands specifying partial purification of the lipase (Figure 2A). Octyl-Sepharose purified fraction showed a maximum specific activity of 72 U/mg and 21-fold purification (Table 1) with a single protein band in SDS-PAGE (Figure 2A).

**Table 1:** Summary of step-wise purification of ERMR1:04 lipase.

**Purification stage**	**Total activity (U)**	**Total protein (mg)**	**Total specific activity (U/mg)**	**Fold purification**	**Yield (%)**
Cell free extract	150	44.0	3.40	1	100
30–80 dialysed	92.25	3.52	26.1	7.6	61.5
Sephadex G-100	40	1.14	35.0	10.2	26.6
Octyl-Sepharose	2.4	0.03	72.7	21.3	1.6

### Determination of Molecular Weight of Purified Lipase and Zymography

The size exclusion and hydrophobic interaction chromatographic purified fractions were analyzed for its molecular weight determination by denaturing SDS-PAGE (12%) and also in non-denaturing Native-PAGE (10%). The size exclusion purified fraction showed multiple bands suggesting partial purification of the protein. The hydrophobic interaction purified protein showed a prominent single band at ~39.8 kDa in denaturing condition (Figure 2A) and 250 kDa in native non-denaturing condition (Figure 2D). The purified ERMR1:04 lipase thus appeared to be hexameric with a monomer unit of 39.8 kDa. The standard curve between log [MW] vs. Rf of proteins moved on SDS-PAGE (12%) was used to determine the exact molecular weight of purified protein (Figure 2C).

Zymogram of the ERMR1:04 lipase showed luminescence under UV in denaturing SDS-PAGE (Figure 2B). This supported that the purified protein is indeed a lipase.

### Peptide Mass Fingerprinting by MALDI-TOF-MS

**Figure 3:**
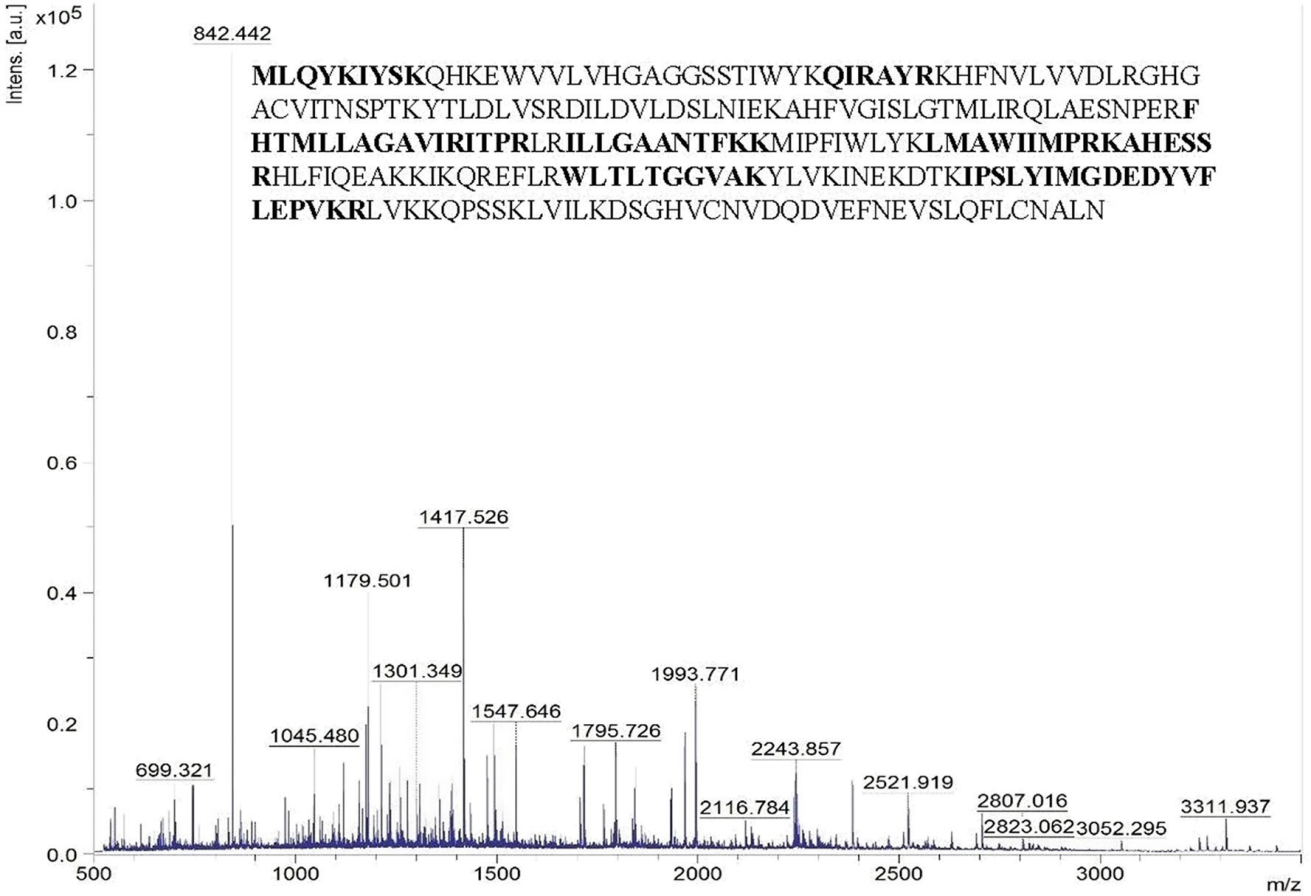
Peptide mass spectra of purified lipase obtained from MALDI-TOF-MS. The x-axis denotes m/z ratio and the y-axis denotes intensity (a.u) of peptides. The amino acid sequence represents the alpha/beta hydrolase of *Solibacillus* sp. to which the query sequence matched. The residues in bold are the ones identical with the query sequence.

The peptide mass pattern obtained after MALDI-TOF-MS analysis followed by MASCOT database search verified that purified protein was a lipase which showed a significant score (130) with the sequence alpha/beta hydrolase (*Solibacillus* sp. R5-41) with 34% sequence coverage (Figure 3). The neighbor-joining tree prepared using the amino acid sequence of alpha/beta hydrolase (topmost MALDI hit) with all the retrieved amino acid sequence of lipase and related proteins of the strain ERMR1:04 showed clustering with GDSL lipase with bootstrap support of 57 (Figure S1). GDSL lipase protein sequence was then used for further *in-silico* analysis.

### Biochemical Characterization of Purified Lipase

#### Effect of Temperature, pH, and Metal Ions

**Figure 4:**
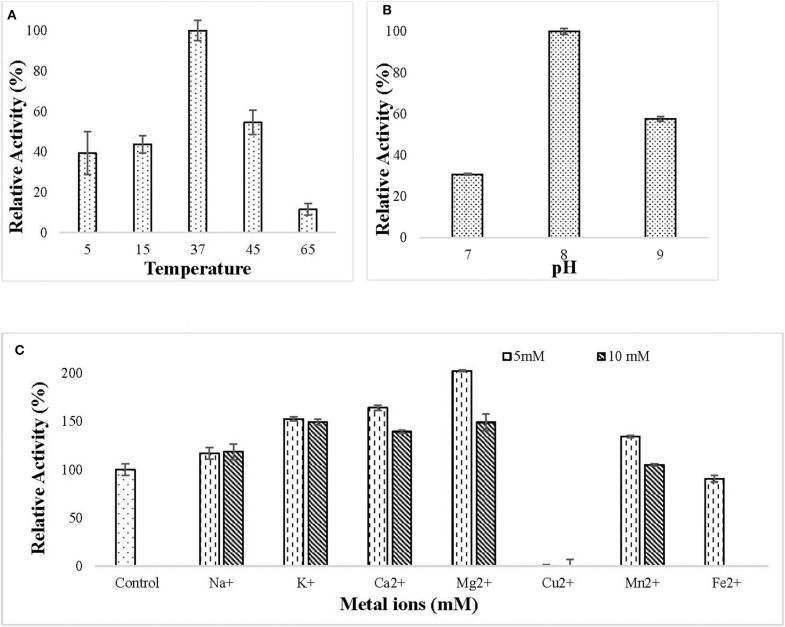
Effect of **(A)** different temperature (5–65°C) on lipase activity. The enzyme assay was performed at a different temperature in 50 mM Tris buffer pH 8. Maximum enzyme activity was observed at 37°C and the enzyme was active in the temperature range of 5–65°C; **(B)** different pH (7–9) on lipase activity. The enzyme assay was performed at different pH in 50 mM tris buffer. Maximum activity was observed at pH 8; **(C)** metal ions (Na^+^, K^+^, Ca^2+^, Mg^2+^, Cu^2+^, Mn^2+^, Fe^2+^) at 5 and 10 mM concentration on lipase activity. The enzyme assay was performed after pre-incubation with metal ions for 30 min at 37°C in 50 mM tris buffer. All the metal ions except (Cu^2+^ and Fe^2+^) increased lipase activity.

The purified lipase was active in the temperature ranging from 5 to 65°C and the optimum temperature for lipase action was observed to be 37°C (Figure 4A). The purified lipase was observed to be active in the pH ranging from 7 to 9 and optimum pH for lipase action was observed to be 8 (Figure 4B). All the tested metal ions except Cu^2+^ and Fe^2+^ enhanced the enzyme activity in both 5 and 10 mM concentration (Figure 4C).

#### Effect of Organic Solvents, Inhibitors, and Kinetic Study of Purified Lipase

**Figure 5:**
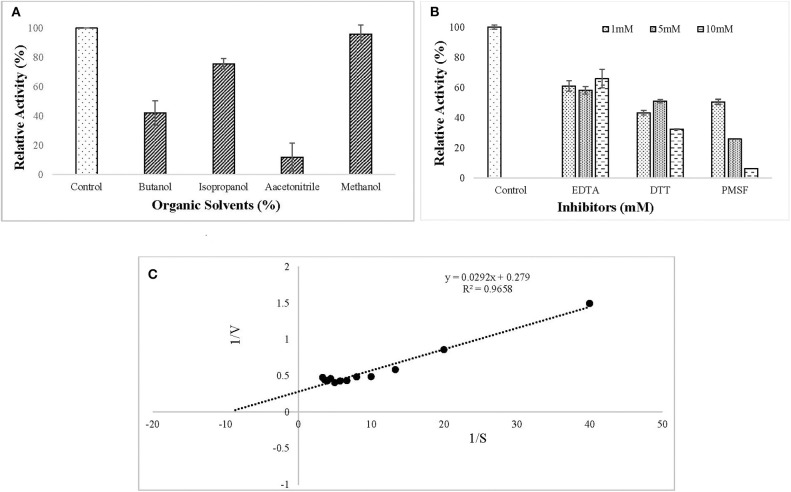
Effect of **(A)** organic solvents (5% (v/v) Butanol, Isopropanol, Acetonitrile, and Methanol) on lipase activity. The enzyme assay was performed after pre-incubation with tested solvents for 30 min at 37°C in 50 mM tris buffer. Lipase retained 80% of activity in isopropanol and methanol while activity was reduced considerably in the presence of butanol and acetonitrile; **(B)** inhibitors (1, 5, 10 mM EDTA, DTT, PMSF) on lipase activity. The enzyme assay was performed after pre-incubation with tested solvents for 30 min at 37°C in 50 mM tris buffer. All the tested inhibitors decreased lipase activity. **(C)** Kinetic study of purified lipase was studied using the Lineweaver–Burk plot. The kinetic parameters (K_m_ and V_max_) were studied by varying *p*NPP concentration from 0.025 to 0.3 mM. The observed K_m_ and V_max_ values were 0.104 mM and 3.58 U/mg, respectively.

The ERMR1:04 lipase retained 80% of activity in isopropanol and methanol while activity was reduced considerably in the presence of butanol and acetonitrile (Figure 5A). All the inhibitors decreased lipase activity. It retained more than 60% of activity in the presence of EDTA and in the presence of reducing agent, i.e., DTT, the residual activity was more than 40%. In the presence of serine protease inhibitor PMSF, the residual activity decreased linearly on increasing the inhibitor concentration in the reaction mixture. The residual activity was 50% in the presence of 1 mM PMSF (Figure 5B). Kinetic parameters, such as K_m_ and V_max_ are a good measure to study substrate affinity and reaction velocity of the purified enzyme. The observed K_m_ value of the purified lipase was 0.104 mM which showed its better substrate affinity. The observed V_max_ value was 3.58 U/mg (Figure 5C).

#### Detergent Compatibility Test

**Figure 6:**
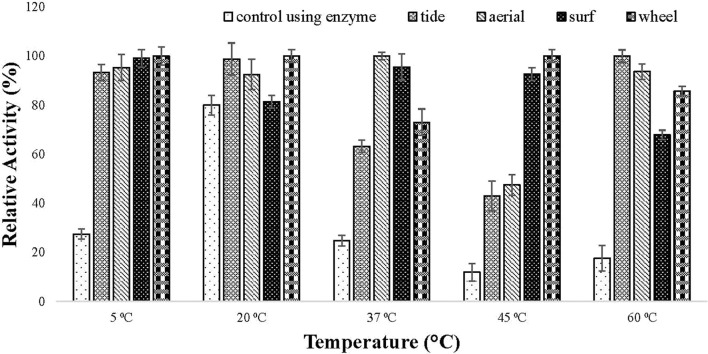
Detergent compatibility test of purified lipase checked on commercially available detergents (1% (v/v) Tide, Surf, Aerial, and Wheel). Lipase assay was performed after incubating the enzyme in the presence of different detergents at a temperature ranging from 5 to 60°C for 30 min in tris buffer. All the detergents showed good compatibility with lipase as the activity increased in the presence of tested detergents.

The compatibility of the enzyme toward different commercial detergents was checked at different temperature. The enzyme showed good hydrolytic activity in all the tested commercial detergents at different temperature. In all the tested detergents, the relative activity of the lipase was higher than the activity of the enzyme alone (Figure 6). This specified that the enzyme is compatible with detergent and can be used in detergent formulations.

### Bioinformatics Analysis

#### Prediction of Lipase Structure and Active Site Analysis

**Figure 7:**
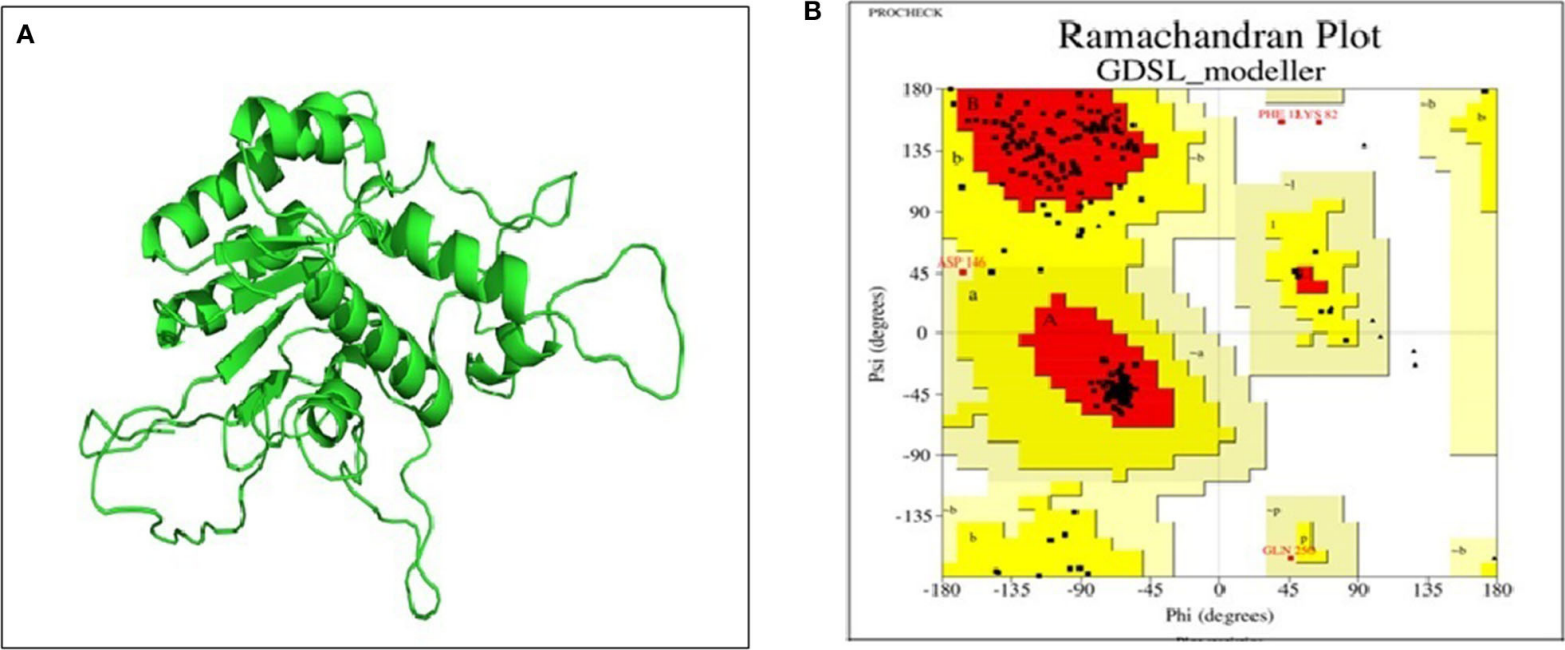
**(A)** 3D model of GDSL lipase built by modeler v9.20; **(B)** model assessment by PROCHECK tool. It describes the quality of the model and shows that 97% region of Ramachandran plot falls in allowed quadrants, thereby specifying a good model quality.

Basic physicochemical parameters calculated by ExPASY tools gave information on the protein sequence, i.e., total amino acid count (281), molecular weight (32623.30 Dalton), iso-electric point (9.39) and the GRAVY index (0.647). For finding templates against protein query, we employed PSI-BLAST rather than simple BLAST to achieve higher accuracy which was failed to achieve by simple BLAST search. From Table S1, 2O14 was the most favorable template based on query coverage, and still, <30% percent identity was achieved. Modeler v9.20 was employed to build a 3D model which used multiple templates instead of a single template for producing a good model prediction. Based on the DOPE score and Z-score in the modeler, the top DOPE score for the best model was −27326.658203 and Z-score was −4.28. This model (Figure 7A) was then further subjected to energy minimization using the steepest descent method. Procheck tool was employed to construct the Ramachandran plot of the protein. The plot (Figure 7B) showed that the constructed model is of good quality as 97% region of Ramachandran plot falls in allowed quadrants. ProSA web server was also used to assess overall model quality based on Z-score which was found to be −5.48 (Figure S2). This suggested a final good model for further downstream bioinformatics analysis. RaptorX web server and COACH tools were employed for exploiting binding site of the studied lipase. The active site in the studied lipase was constituted by Ile41, Ile92, Tyr130, Ile173, Pro210, Tyr211, Phe272, Val275, Thr278, Gln279, and Lys280 residues.

#### Molecular Docking of Lipase Using Autodock and Simulation Analysis

**Figure 8:**
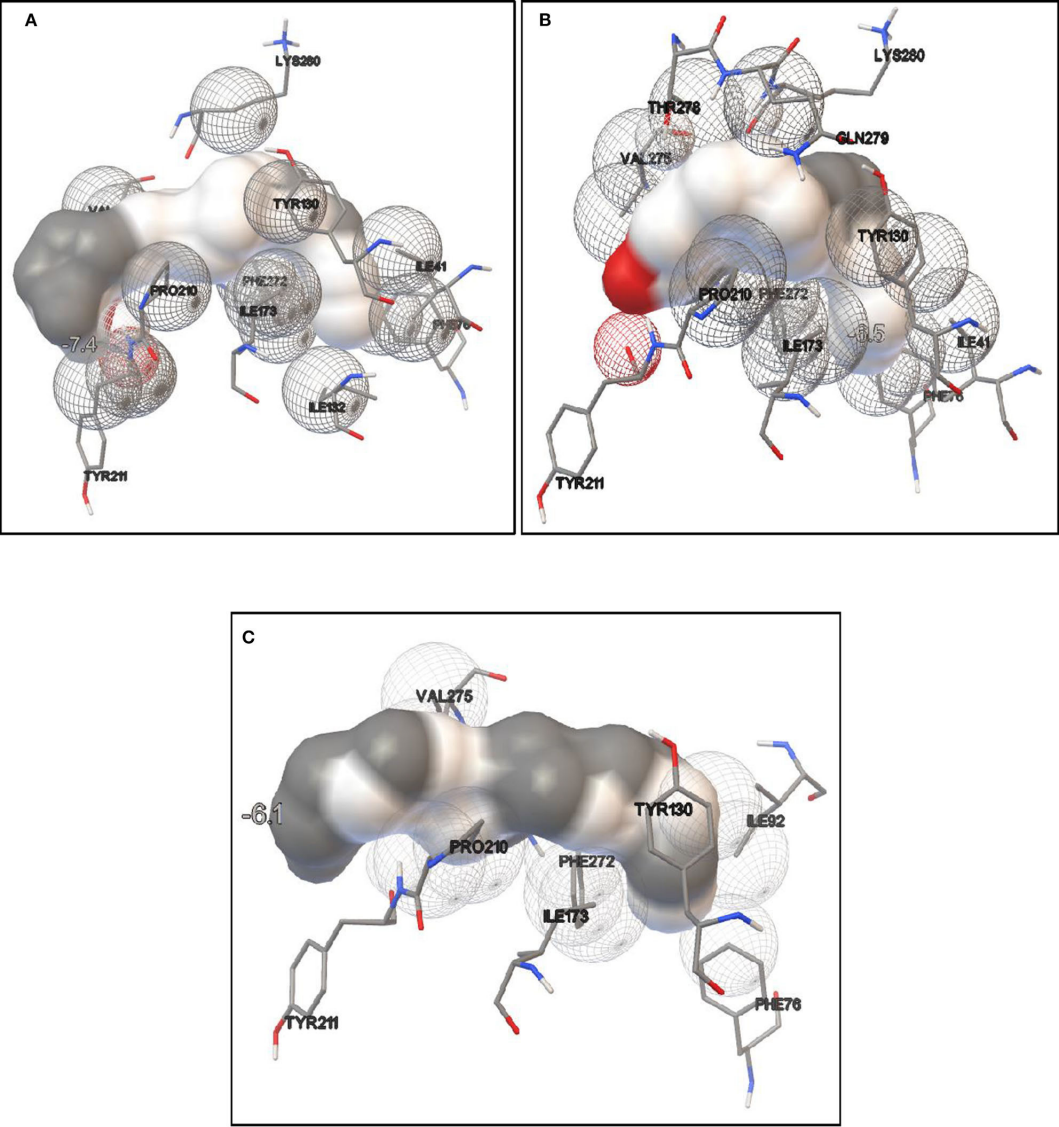
Molecular docking simulation study of lipase with different fatty acids. It is used to predict the binding affinity of the enzyme toward different fatty acids which acts as inducers in lipase production. **(A)** Docking study in the presence of linoleic acid. The binding energy is −7.4 kcal/mol; **(B)** docking study in presence of palmitic acid, the binding energy is −6.5 kcal/mol; **(C)** docking study in presence of oleic acid, the binding energy is −6.1 kcal/mol. It is observed that maximum binding energy is observed in the presence of linoleic acid so it acts as a better inducer for ERMR1:04 lipase production.

**Figure 9:**
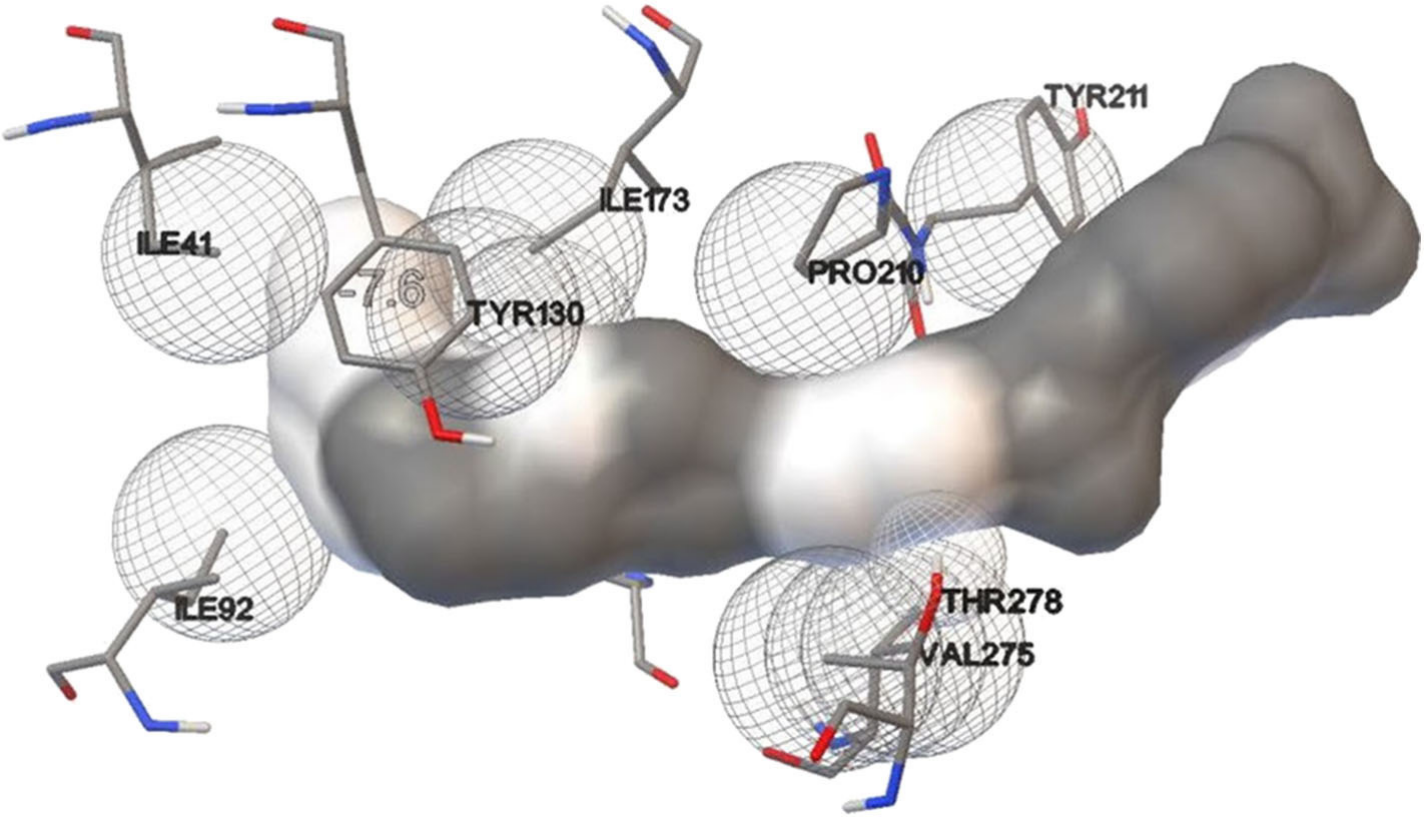
Molecular docking simulation study of lipase with substrate *p*-nitrophenyl palmitate. It is used to predict the interaction pattern between the active site of protein and ligand. The binding affinity for *p*-nitrophenyl palmitate is (−7.6 kcal/mol), thereby specifying a good interaction with the lipase.

Lipase protein and ligand (given substrates) prepared in Autodock tools according to the protocol grid box dimensions were adjusted to 40 × 46 × 40 with 0.580 Å spacing. Effect of different inducers (linoleic acid, palmitic acid, and oleic acid) was checked to know the effect of different oils on lipase production. From the study, it was observed that linoleic acid had a maximum binding affinity (−7.4 kcal/mol) toward the enzyme, followed by palmitic acid (−6.5 kcal/mol) and oleic acid (−6.1 kcal/mol) (Figure 8). So, linoleic acid followed by palmitic acid acts as the best inducer for lipase production than the other tested fatty acids in ERMR1:04. To know the nature of the enzyme various substrates were used for docking study. Among three tested substrates, *p*-nitrophenyl palmitate showed the best binding affinity with the top score of −7.6 kcal/mol (Figure 9). Other substrates *viz*., *p*-nitrophenylphosphoryl choline interacts with −7.0 kcal/mol binding affinity and prime amino acid residues were Ile92, Pro210, Phe272, Val275, and Thr278 also shown in Figure S3. *p*-nitrophenyl acetate interacted with least energy of −6.3 kcal/mol depicted in Figure S4.

**Figure 10:**
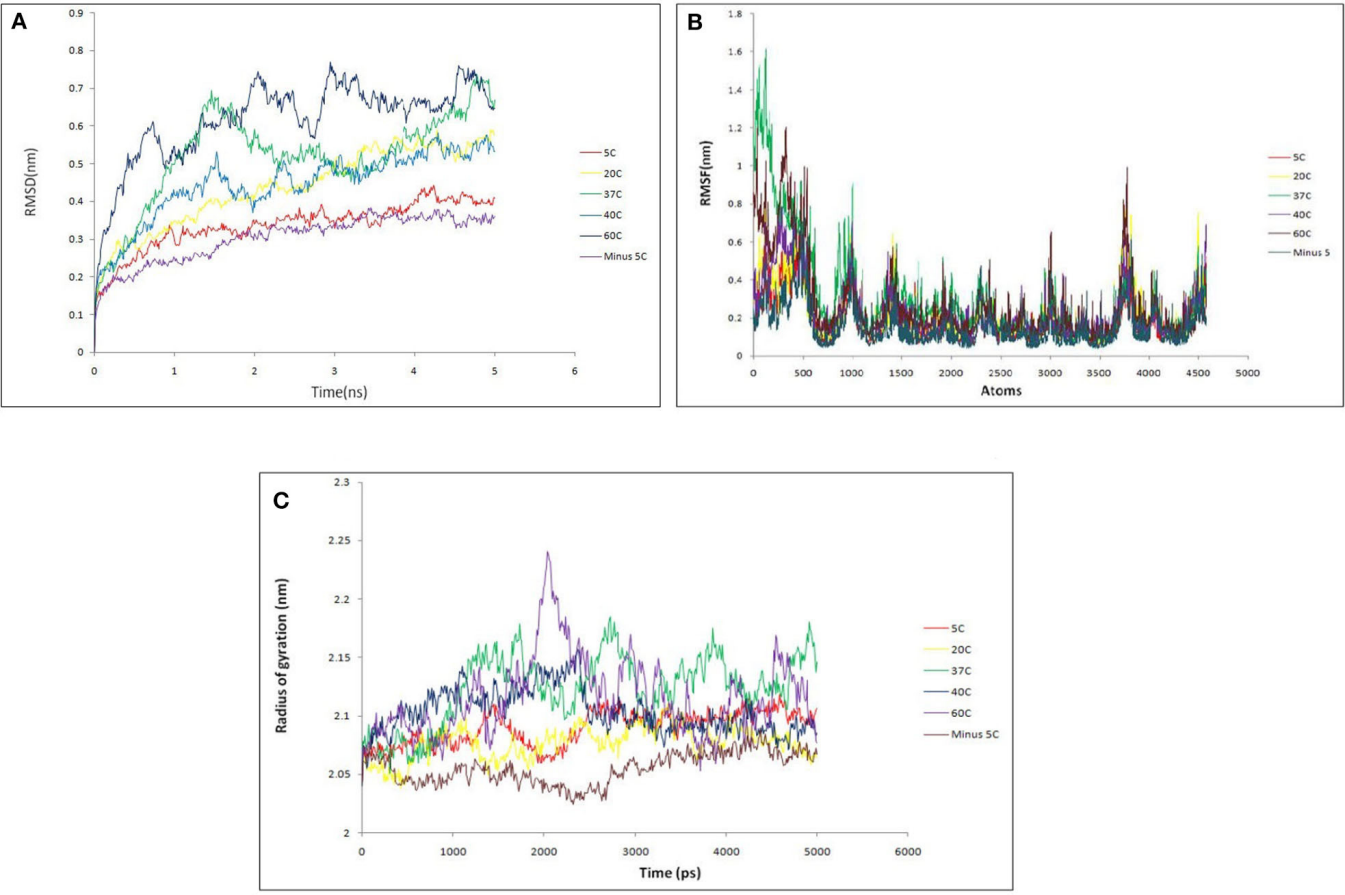
**(A)** Backbone RMSD comparison of lipase protein at various temperature (−5, 5, 20, 37, 40, 60°C). It shows that protein remains stable throughout 5 ns with insignificant fluctuation in protein backbone at 60°C. The protein did not break down at any interval across the time; **(B)** RMSF comparison of lipase protein at various temperature; **(C)** radius of gyration comparison of lipase protein at various temperature. It shows the minor fluctuation in lipase structure at 60°C over 2 ns but overall protein structure remains stable.

Then, Molecular dynamics studies (MDS) was carried out for lipase alone as well as for lipase in complex with substrates at various temperature to understand the broad character of the enzyme. The intact protein was simulated at 268.15 K (−5°C), 278.15 K (5°C), 293.15 K (20°C), 310.15 K (37°C), 313.15 K (40°C), and 333.15 K (60°C) temperature. Various parameters like RMSD, RMSF, and radius of gyration were considered to assess the stability of lipase protein. The overall RMSD plot (Figure 10A) showed that protein remains stable throughout 5 ns with insignificant fluctuation in protein backbone at 333.15 K (60°C), although the protein did not break down at all intervals across the periods. RMSF (Figure 10B) and Radius of Gyration (Figure 10C) plots also supported a similar observation as that of RMSD results. Lipase protein reached maximum stability at 268.15 K (−5°C) and minute fluctuations at 333.15 K (60°C), showing significantly fluctuations with the increasing temperature but maintaining the overall compactness. The backbone RMSD plot in the presence of substrates showed good compactness in protein structures with mild fluctuations (Figure S5).

## Discussion

Psychrotrophic organisms possess cold-adapted proteins and enzymes which help them to maintain their metabolic function in low temperature. In the present study, an extracellular cold-adapted lipase enzyme was purified and characterized from an efficient lipase producing psychrotrophic bacterium *C. polytrichastri* ERMR1:04 isolated from East Rathong glacier, Sikkim Himalaya.

It is known that the enzyme production is greatly dependent on the culture conditions like pH, temperature, and substrate types. In this study, maximum lipase production was observed in the pH range of 7–8, which specified that neutral to alkaline pH condition favors the lipase production by *C. polytrichastri* ERMR1:04. The growth optima for the organism was also observed in the pH range of 6–10. Similar findings on lipase production in neutral to alkaline media conditions by psychrotrophic bacteria isolated from alpine regions are reported before (Barbaro et al., 2001; Gupta et al., 2004; Joseph and Ramteke, 2013; Salwoom et al., 2019b). The optimum temperature for enzyme production depends on the growth temperature of the organism. *C. polytrichastri* ERMR1:04 grows well at a temperature range of 4–28°C. The enzyme production was maximum at 20°C, and a drastic decline in the production was observed with increasing temperature. Lipase is an inducible enzyme, induced by oils or fats in the culture medium (Bisht et al., 2013). In the current study, like its inherent property, lipase production was observed in all the tested lipids. The maximum production was observed in rice bran oil followed by olive oil. The major fatty acid composition of rice bran oil includes oleic acid (18:1), linoleic acid (18:2), and palmitic acid (16:0) (Zullaikah et al., 2005). Different oils differ with the proportion of these fatty acids. To predict the reason behind the preference of rice bran oil as an inducer for lipase production in ERMR1:04, binding affinities of the enzyme toward oleic acid, linoleic acid, and palmitic acid were checked by molecular docking study. The ERMR1:04 lipase showed maximum affinity toward linoleic acid, followed by palmitic acid and oleic acid. Among all the tested oils, the affinity results suit the best with rice bran oil as after oleic acid, the highest percentage of composition is linoleic acid and palmitic acid. Like, in case of olive oil, the highest composition is of oleic acid but it has very low percentage of linoleic and palmitic acid (Benitez-Sánchez et al., 2003). Hence, it is hypothesized to be due to the affinity potential of the purified enzyme toward linoleic and palmitic acids, that the superior induction and production of ERMR1:04 lipase is shown in the presence of rice bran oil. Earlier also, it has been reported that the fatty acid profile of oils affects lipase production by an organism (Lakshmi et al., 1999; Wang et al., 2008; Darvishi et al., 2009). Rice bran oil is low priced and readily available and the ability of the strain to produce lipase on cheap carbon source is advantageous to reduce the enzyme production cost. In addition to the carbon source, the type of nitrogen source also affects extracellular lipase production. Generally, the organic form of nitrogen, such as peptone or yeast extract is preferred for lipase production (Ghanem et al., 2000; Sharma et al., 2002; Salwoom et al., 2019b). Lipase production by ERMR1:04 was done in the presence of yeast extract as a nitrogen source which is in favor of previous findings.

Purification of lipase from the culture supernatant itself is a tedious task but a certain degree of purification is necessary depending on the application of enzyme (Joseph et al., 2008). The purification process removes contaminants and improves stability, activity and shelf life of the enzyme. Furthermore, purified protein is required for studying structure and conformation, the kinetic and thermodynamic mechanisms for substrate hydrolysis, and structure-function relationships (Javed et al., 2018). In current work, the crude enzyme was successfully purified to homogeneity in two steps, i.e., gel filtration and hydrophobic interaction chromatography with 21-fold purification and a 1.6% yield. There are numerous studies on multi-step purification of cold-active lipase from various psychrophilic and psychrotrophic bacteria, where an ammonium salt precipitation in the early stage is followed by a combination of chromatographic steps (Wang et al., 2012; Li et al., 2013; Bae et al., 2014; Ji et al., 2015). The lower yield of the enzyme might be due to the combined use of two chromatographic steps and loss of protein during purification (Li et al., 2013; Bae et al., 2014; Ji et al., 2015). The purified lipase appeared to be a 250 kDa hexameric protein with a monomer unit of 39.8 kDa as confirmed by the Native and SDS PAGE, respectively. Zymography in the presence of substrate MUF butyrate supported the identity of the purified protein as a lipase. Further, the protein sequence generated using MALDI-TOF-MS of the purified lipase showed maximum similarity with the alpha/beta hydrolase a superfamily of lipase. Afterwards, lipase and related protein sequences were retrieved from *C. polytrichastri* ERMR1:04 draft genome and then a phylogenetic tree was constructed between those retrieved protein sequences and MALDI-TOF-MS topmost hit, i.e., alpha/beta hydrolase. The phylogenetic tree showed clustering of alpha/beta hydrolase with GDSL family lipase of strain ERMR1:04, which was further used for structural analysis by homology modeling.

Currently, homology modeling is the most commonly used computational approach to predict and generate structural models of biological macromolecules like proteins. In the present study, an orthologous approach was used to build a 3D structure of the amino acid sequence of the purified lipase using modeler v9.20. The 3D structure was significantly valid and supported by well-known parameters like ProSA web server, Ramachandran plot and Q mean score. Previously, homology modeling approach has been used successfully to predict the 3D structure of several cold-active lipases produced by psychrotrophic bacteria (De Pascale et al., 2008; Xu et al., 2010; Kamarudin et al., 2014; Jalil et al., 2018). The final 3D model obtained was further exploited for finding the active site, docking and MD simulation studies. While analyzing ERMR1:04 domain architecture, the domain length stretch expands between 39 and 271 amino acid residues according to NCBI-CDD and those active site residues were located outside of the domain length (Phe272, Val275, Thr278, Gln279, and Lys280). This finding revealed a broad substrate specificity of ERMR1:04 lipase. Docking results also supported the good interaction between amino acid residues outside of the lipase domain and substrates.

Biochemical characterization of the purified lipase showed its activity in the pH ranging from 7 to 9 with an optimum activity at pH 8. This specified that the purified lipase is alkaline. Lipases with activity in a pH range of 7.6–8.6 with an optimum at 8 (Jinwal et al., 2003), pH range 8.0–10.5 with an optimum at 8.5 (Kumar et al., 2005) and pH range 7–9 with an optimum at 8.5 (Joshi et al., 2006) have been defined as alkaline in previous studies. Bioinformatics analysis performed by using the amino-acids sequence of purified lipase showed that the isoelectric point of the purified lipase is 9.39, thereby confirming the alkaline nature of the enzyme. Alkaline lipases are important in many industrial applications like leather processing, detergent formulations and sewage treatment (Salwoom et al., 2019b). The ERMR1:04 lipase was active over a broad temperature range of 5–65°C, with 37°C as the optimum. This property can be valuable in many industrial processes which require low to high temperature. Most of the cold-active lipases isolated and purified till now doesn't show activity at a higher temperature (Joseph et al., 2012; Kryukova et al., 2019; Li et al., 2019; Salwoom et al., 2019a). The lipase isolated in the current study is remarkably active in a broad temperature range of 5–65°C and could be a valuable asset in many industrial processes like in detergent formulations. The lipase producing bacterium in the current study has been isolated from East Rathong Glacier, Sikkim Himalaya where temperature fluctuations are common, ranging between −20 and 5°C (Agrawal and Tayal, 2015). Being subjected to this kind of broad temperature fluctuation, the psychrotrophic bacteria from such an environment are likely to produce versatile enzymes with broad temperature activity. In previous studies, a cold active metalloprotease enzyme isolated from a glacier bacterium (Margesin et al., 2005) and a protease isolated from a bacterium of a glacier at Lahaul and Spiti, India (Salwan et al., 2010) showed broad temperature activity. Most enzymes are metalloenzymes whose activity increases in the presence of specific metals. In this study, the lipase activity increased in Na^+^, K^+^, Ca^2+^, Mg^2+^, and Mn^2+^ ions. This showed that the enzyme is metal activated, whereby the metal ions increase the stability of the enzyme structure by changing its conformation to a stable one (Rahman et al., 2005). Other metal ions, such as Cu^2+^ and Fe^2+^ slightly inhibited the activity, indicating that these metal ions are altering the enzyme structure (Lee and Rhee, 1993; Kambourova et al., 2003; Joseph and Ramteke, 2013). Kinetic study of the purified lipase showed Km of 0.104 mM and Vmax of 3.58 U/mg for *p*NPP. This specified that the purified lipase has a decent substrate specificity and activity toward C-16 acyl group esters, thereby confirming the lipolytic nature of the purified enzyme as lipases show maximum activity toward long-chain esters. All the tested enzyme inhibitors reduced the lipase activity, and in the presence of serine protease inhibitor PMSF, a linear decrease in the residual activity was observed on increasing the inhibitor concentration in the reaction mixture. This indicated that the enzyme has active sites resembling the serine protease. The organic solvents (5%; v/v) butanol and acetonitrile inhibited the lipase activity while isopropanol and methanol were fairly tolerated by the enzyme. This might be caused by the denaturing effect of butanol and acetonitrile on the protein.

Proteins, starch and lipids are the main components of dirt present in clothes (Olsen and Falholt, 1998). The stains present in the clothes are generally removed by beating and heating of the fabrics in presence of detergents which further shorten the life of fabrics, and is an energy-requiring process (Al-Ghanayem and Joseph, 2020). Addition of lipolytic enzymes in the detergents is becoming a common practice to improve the effectiveness of the detergent (Hasan et al., 2006). In cold environments, water used in washing should be heated for the proper functioning of enzymes as most of the mesophilic enzymes are inactive at low temperature (Al-Ghanayem and Joseph, 2020). So cold-active enzymes would be beneficial for detergents at cold regions (Joseph et al., 2008). On the other hand, at a higher temperature, most of the cold-active enzymes become inactive, so the use of a thermostable enzyme would be beneficial (Zamost et al., 1991). To address these problems, the introduction of an alkaline, cold-active enzyme with broad temperature activity as a detergent additive would be beneficial (Maharana and Ray, 2015). The lipase purified in the current study would be a better choice to use in the detergent formulation as it is active in alkaline pH ranging from 7 to 9 with optimum activity at pH 8 and temperature range of 5–65°C with an optimum at 37°C. Lipases with activity in the pH range of 7–9 with an optimum at 7 and temperature range of 0–60°C with an optimum at 15°C (Maharana and Ray, 2015), pH range of 7–11 with an optimum at 8.5 and temperature range of 5–37°C with optimum at 20°C (Li et al., 2014) were suggested as good detergent additives in previous studies. The compatibility of the purified enzyme with common commercial detergents was checked to see its effectiveness. The activity of purified lipase increased by ~50% in the presence of all the tested commercial detergents at a temperature ranging from 5 to 60°C. In earlier studies, it has been hypothesized that enhancement of lipase activity is due to better accessibility of the substrate molecules. Binding of detergent at the active sites of the enzyme leading to its conformational change has been reported to be the possible reason for such improved substrate accessibility (Helistö and Korpela, 1998; Salameh and Wiegel, 2010). The increase in the activity of ERMR1:04 lipase in presence of commercial detergents is in agreement with several earlier lipase studies (Hemachander and Puvanakrishnan, 2000; Cherif et al., 2011; Chauhan et al., 2013; Phukon et al., 2020; Sahoo et al., 2020). The studied lipase with activities in a broad temperature and alkaline pH proves to be a suitable detergent additive for enhanced washing performance at different environments ranging from colder to moderately high-temperature regions.

## Conclusion

In the present study, a cold-adapted, broad temperature active lipase was extracted and purified to homogeneity from *C. polytrichastri* ERMR1:04. The molecular weight of the monomeric unit of purified lipase was 39.8 kDa and it was a 250 kDa hexameric protein. Peptide mass fingerprinting of the purified protein confirmed it as lipase showing maximum similarity with alpha/beta hydrolase (lipase superfamily). The purified protein was broad temperature active (5–65°C) and alkalophilic. Bioinformatics predictions supported the cold-adapted and broad temperature activity of our lipase. Additionally, the *in-silico* docking studies predicted diverse substrate specificity of the purified lipase. The lipase showed promising detergent stability in presence of common commercial detergents. Owing to its versatile temperature and substrate range, and its enhanced activity with commercial detergents, the lipase qualifies as a potential candidate in detergent formulations. To the best of our knowledge, this is the first study on purification and characterization of lipase enzyme from any species of the genus *Chryseobacterium*.

## Data Availability Statement

The datasets presented in this study can be found in online repositories. The names of the repository/repositories and accession number(s) can be found in the article/Supplementary Material.

## Author Contributions

AK: conceptualization, methodology, validation, investigation, writing—original draft, and visualization. SM: methodology, investigation, and writing—original draft. NK: software, formal analysis, investigation, and writing—original draft. VA: resources, writing—review and editing, and project administration. SK: project administration and funding acquisition. RK: conceptualization resources, writing—review and editing, supervision, project administration, and funding acquisition. All authors contributed to the article and approved the submitted version.

## Conflict of Interest

The authors declare that the research was conducted in the absence of any commercial or financial relationships that could be construed as a potential conflict of interest.
