# Supplementary material for: A Broad Temperature Active Lipase Purified From a Psychrotrophic Bacterium of Sikkim Himalaya With Potential Application in Detergent Formulation
Source: Front Bioeng Biotechnol. 2020 Jun 25;8:642. doi: 10.3389/fbioe.2020.00642 (PMC7329984; doi:10.3389/fbioe.2020.00642)
Supplement: Supplementary file 1 [file Data_Sheet_1.docx]

**A broad temperature active lipase purified from a psychrotrophic bacterium of Sikkim Himalaya with potential application in detergent formulation**

Anil Kumar, Srijana Mukhia, Neeraj Kumar, Vishal Acharya, Sanjay Kumar, Rakshak Kumar


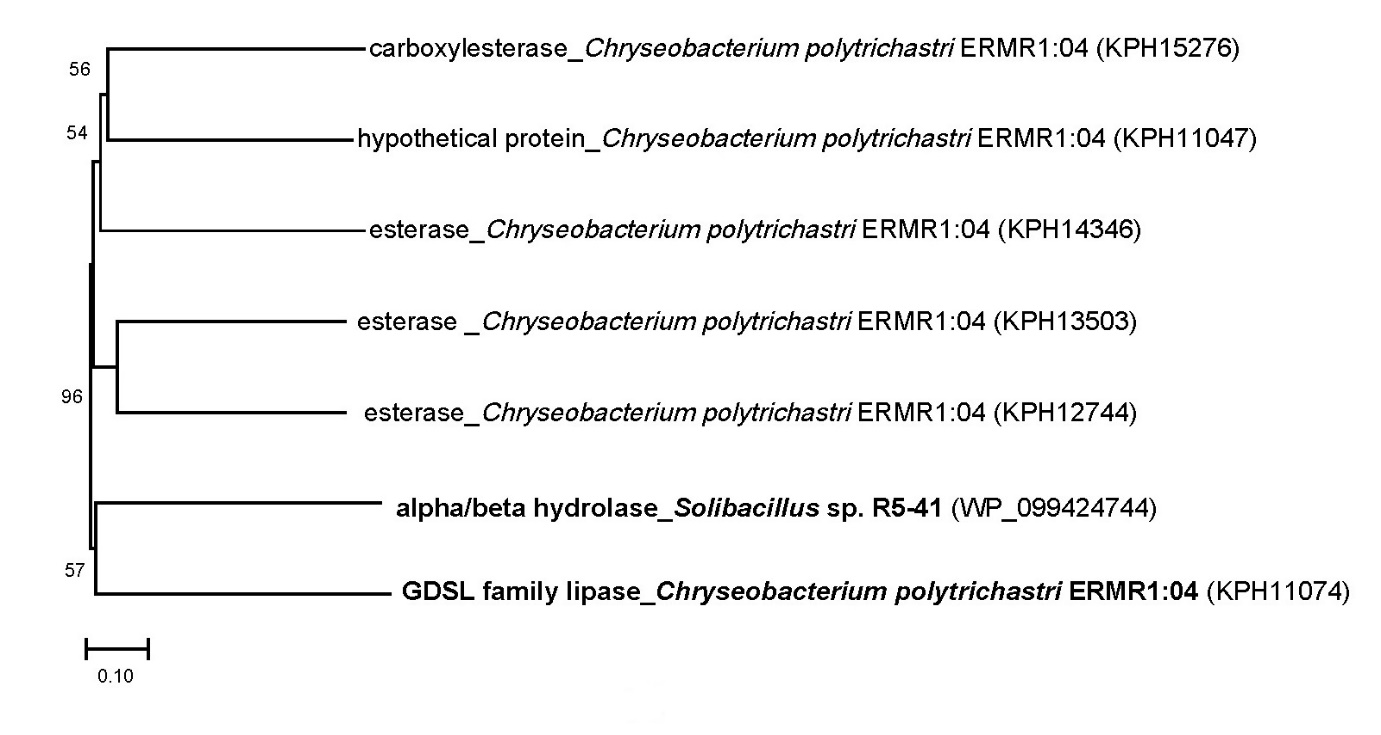


**Fig. S1** Phylogenetic tree based on the topmost MALDI hit alpha/beta hydrolase of *Solibacillus* sp. R5-41 (obtained from MASCOT database) and lipase and related protein sequences of *C.* *polytrichastri* ERMR1:04 (retrieved from the draft genome using NCBI database). The sequences were aligned using ClustalW and the neighbour-joining tree was constructed using the p-distance method of amino acid substitution with 1000 bootstrap replications. Bootstrap support values are depicted above branches. Values that were not higher than 50% are not shown. The scale bar corresponds to the number of amino acid differences per site. NCBI accession numbers are given in parenthesis. The *C.* *polytrichastri* ERMR1:04 lipase sequence clustering with the *Solibacillus* sp. alpha/beta hydrolase is shown in bold.


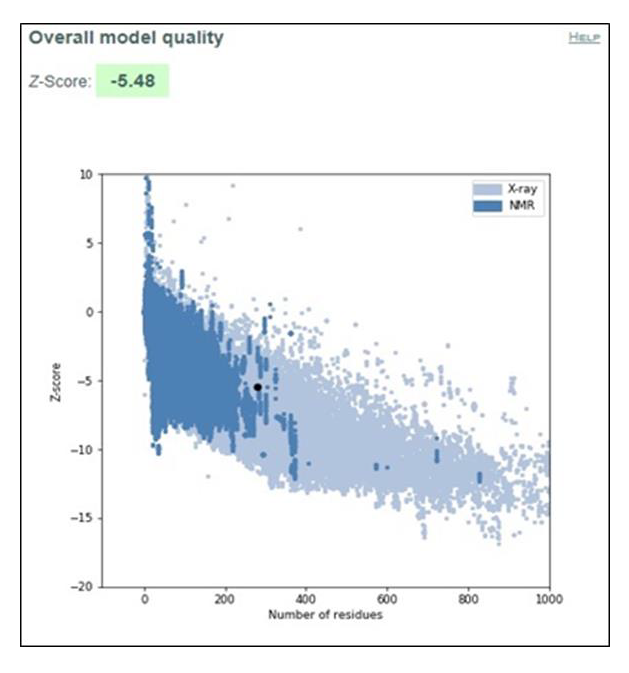


**Fig. S2** Model assessment by ProSA web server with Z-score. It describes the quality of the model and shows the Z-score of -5.48, thereby specifying a good model quality.


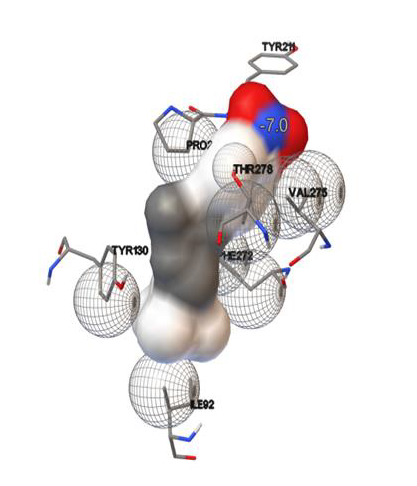


**Fig. S3** Molecular docking simulation study of lipase with substrate *p*-nitrophenylphosphoryl choline. It is used to predict the interaction pattern between the active site of protein and ligand. The binding affinity is ( -7.4 kcal/mol), thereby specifying a good interaction with the lipase.


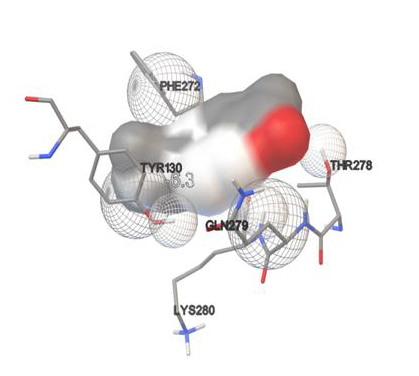


**Fig. S4** Molecular docking simulation study of lipase with substrate *p*-nitrophenyl acetate. The binding affinity is ( -7.0 kcal/mol), thereby specifying a good interaction with the lipase.


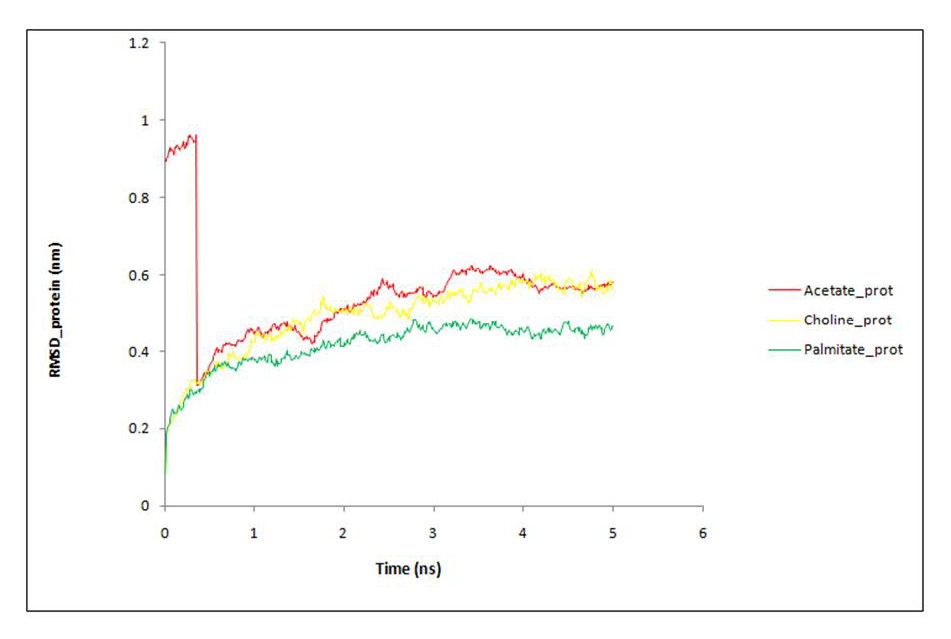


**Fig. S5** Backbone RMSD comparison of lipase protein with various substrates. The backbone RMSD plot in the presence of substrates showed good compactness in protein structures with mild fluctuation.

**Table S1:** PSI-BLAST output with parameters.

| **PDB ID** | **E - value** | **Percent Identity** | **Query Coverage** |
| --- | --- | --- | --- |
| 2O14 | 5e-18 | 26.91% | 95% |
| 1DEO | 1e-11 | 26.46% | 60% |
| 3C1U | 2e-11 | 26.98% | 60% |
| 2LY8 | 3e-05 | 31.58% | 25% |
